# Knowledge, Attitude and Practices of Swallowing and Dysphagia: A Cross‐Sectional Survey Among the Community Dwelling Adults of South India

**DOI:** 10.1111/1460-6984.70273

**Published:** 2026-06-12

**Authors:** Thejaswi Dodderi, Snezan Wayne Dsouza, Viola Maryann Dsouza, Radish Kumar Balasubramanium, Mohit Kothari

**Affiliations:** ^1^ Department of Audiology and Speech Language Pathology Kasturba Medical College Mangalore, Manipal Academy of Higher Education Manipal India; ^2^ Nitte (Deemed to be University) Nitte Institute of Speech and Hearing (NISH) Mangalore India; ^3^ Hammel Neurorehabilitation Center and University Research Clinic Department of Clinical Medicine, Aarhus University Hammel Denmark; ^4^ Department of Clinical and Preventive Odontology, Faculty of Health Sciences University of Southern Denmark Odense Denmark

**Keywords:** awareness, community, dysphagia, health, swallowing

## Abstract

**Background:**

Community awareness of swallowing and dysphagia plays a significant role in improving early‐referral behavior. Literature reports that community awareness of swallowing and dysphagia in the Western countries to be low. Conversely, India has a diverse cultural, linguistic and healthcare‐related factors which may influence the health‐seeking behavior and early‐referral of dysphagia. Hence, it becomes difficult to generalize the findings of existing literature.

**Aim:**

The primary objective of this study was to analyze the knowledge, attitudes and practices (KAP) related to swallowing and dysphagia among the community dwelling adults of South India. The secondary objective was to evaluate if age, sex and education levels were associated with these factors.

**Methods and Procedures:**

Community dwelling adults were recruited from Mangalore city, India for a cross‐sectional survey using a convenience sampling design. A purposive built content validated 27‐item questionnaire was distributed using Google Form and/or hard copies to the participants for data collection. Participant's responses were independently analyzed by two Speech Language Pathologists using open content analysis. The responses were summarized as percentages and categories and chi square test was administered to examine the association between age, sex and education levels and KAP enquiries at 0.05 level.

**Outcomes and Results:**

A total of 372 participants aged 20–79 years (mean age = 46.26; SD = 15.63) participated in the study. Participants demonstrated limited knowledge of signs, causes, complications and professional management of dysphagia. Aspiration was widely recognized as unsafe, yet only one‐tenth correctly identified pneumonia as a complication of dysphagia. Less than one‐fifth knew the treatment options for dysphagia. Attitudes reflected fear of choking, discomfort towards nasogastric‐tube feeding and moderate willingness to seek help from healthcare professionals. Practices suggested low awareness of Heimlich maneuver, inconsistent food‐texture modification and reliance on water to manage dry mouth. Age, sex and education levels were significantly associated with knowledge (saliva is important for chewing, avoid eating in public space to overcome stigma, early intervention helps regain swallowing abilities, age range frequently associated with dysphagia), attitude (fear of choking, preference to non‐oral feeds), practices (using internet to find solutions for dysphagia, drinking water before meals or moistening food using water, changing food consistencies, using straw over cup) at *p* > 0.05 level.

**Conclusions and Implications:**

Community dwellers demonstrated insufficient knowledge, predominantly negative attitudes and unsafe self‐management practices related to swallowing and dysphagia. Strengthened public education, early‐referral pathways and inclusion of basic life‐support skills are highly warranted to improve community awareness on swallowing and dysphagia.

**WHAT THIS PAPER ADDS:**

*What is already known on this subject*

- Community awareness of dysphagia is low in Western countries. Cultural beliefs, literacy, superstition and access to healthcare influence what people know, how they respond and when they seek help. Understanding knowledge, attitude and practices (KAP) of swallowing and dysphagia in India is critical for reducing self‐management risks and improving early‐referral behavior.

*What this study adds to existing knowledge*

- The study provides the first structured KAP analysis on swallowing and dysphagia in an Indian community . Participants recognized aspiration as dangerous, yet lacked awareness of critical signs, causes, treatment options and responsible team members. Fear of choking was high, and most were uncomfortable with tube‐based feeding. The public seldom employed safe compensatory strategies and very few were familiar with Heimlich maneuver or food‐texture modification. Age, sex and education levels were significantly associated with multiple KAP variables.

*What are the clinical implications of this work?*

- There is an urgent need to conduct more public awareness programs on prevention, early identification and intervention of dysphagia by healthcare professionals involved in dysphagia care. Efforts must be scaled to provide basic life support (BLS) skills training programs, including training in Heimlich maneuver, to the public.

## 1 Introduction

Dysphagia, or difficulty swallowing, is a pressing global health concern, affecting approximately 43.8%–60% of adults (Rajati et al. 2022, Ribeiro et al. 2023). Dysphagia is a common sequela that poses a challenge for adults who have had a stroke, progressive neurogenic diseases and head and neck cancer, among others (Groher and Crary 2010, Roden and Altman 2013). In India, it is estimated that 47.7% of stroke patients will experience dysphagia, with over half of them developing aspiration pneumonia (Krishnamurthy et al. 2022). Moreover, the older adult population is particularly vulnerable to dysphagia (Baijens et al. 2016, Madhavan et al. 2016, Namasivayam‐MacDonald et al. 2023), with 36.6% of community dwelling older adults in Mangalore, India self‐reporting swallowing difficulties (Dodderi et al. 2024). Individuals with dysphagia encounter two functional difficulties: (1) swallowing safely, without penetration and/or aspiration, and (2) swallow efficiently, without leaving residue in the upper airway. Aspiration pneumonia is one of the serious health consequences of dysphagia (Martino et al. 2005, Smithard et al. 1997). Unresolved dysphagia is significantly associated with low nutrition and hydration levels (Wirth et al. 2016), disruption of eating and drinking‐related activities (Ekberg et al. 2002), lower overall quality of life (Jones et al. 2018) and mortality (Martino et al. 2005). As a collective health issue, dysphagia contributes to adverse health outcomes, resulting in prolonged hospital stays and increased medical costs (Dziewas et al. 2021).

Dysphagia, despite its significant ramifications on health and swallowing outcomes, often remains neglected and under‐reported among healthy (Leslie and Smithard 2021) and clinical populations (Ekberg et al. 2002, Pauloski et al. 2002). This issue can be attributed to the lack of public awareness about the signs and symptoms of dysphagia (McHutchion et al. 2021, Molfenter et al. 2025, Pu et al. 2025) and subjective experience of swallowing often does not align with clinical assessment findings (Namasivayam‐MacDonald et al. 2019, Pu et al. 2025). For example, one study reported that 59.25% of individuals with stroke were unaware of their dysphagia symptoms (Parker et al. 2004). Similarly, individuals with Parkinson's disease often self‐reported voice and swallowing difficulties only ten years after the onset of the disease (Silbergleit et al. 2021). Interestingly, one in six adults experiences dysphagia, but only half of them seek treatment (Adkins et al. 2020). Further, a recent study revealed that many symptoms of swallowing difficulties, such as nasal regurgitation, choking and coughing, were reported in healthy adults who passed the swallowing screening checklist Eating Assessment Tool‐10 (Leslie and Smithard 2021). Collectively, the literature suggests that swallowing difficulties are neglected and individuals become concerned about dysphagia only when swallowing difficulties disrupt their day‐to‐day activities. Hence, addressing dysphagia requires collective efforts from individuals with dysphagia, their family members, and society to enable early identification and intervention of dysphagia and alleviate its burden.

Public awareness of signs and symptoms or risk factors plays a significant role in encouraging early self‐referral for any medical issues, particularly dysphagia. Therefore, surveying the knowledge, attitude and practices (KAP) related to swallowing and dysphagia can shed light upon prevalent myths, misconceptions and unhealthy self‐practices and behaviors that have developed within society and carried forward over time (Jacobsen 2016). Additionally, studying KAP in the 21st century is crucial, as the public often relies upon the Internet as the primary source of information, which can be unreliable, conflicting and misleading (Chang et al., 2022; Chang and Park, 2021). Furthermore, studies on KAP related to swallowing and dysphagia among community dwellers are increasing (Pu et al. 2025). Existing research in the West indicates that community dwellers generally possess low knowledge about causes, signs and symptoms, evaluation and treatment of dysphagia (McHutchion et al. 2021, Molfenter et al. 2025). It is important to note that cultural beliefs, customs and superstitions significantly influence KAP of medical conditions, making it essential to contextualize findings to specific regions like India. By studying KAP among the community dwellers, we gain valuable insights into internal barriers and facilitators that affect self‐referral of dysphagia. Further, examining KAP aligns with Goal 3 of the Sustainable Developmental Goal of the United Nations (United Nations n.d.), which focuses on good health and well‐being.

In the present study, we investigated the following research objectives to understand better the KAP related to swallowing and dysphagia among community dwelling adults of South India.

1. Examine public knowledge of swallowing and dysphagia.
2. Profile attitudes towards dysphagia and individuals experiencing swallowing difficulties.
3. Document common practices followed to overcome swallowing difficulties.
4. Evaluate whether age, sex and education levels were associated with KAP parameters.

## 2 Materials and Methods

### 2.1 Survey Design

The present study was undertaken in Mangalore, a coastal metropolitan city in Southern part of India. A cross‐sectional design was implemented in the study. The Institute Ethics Committee of the first author's institution sanctioned ethical clearance to conduct the study. Before collecting data, all participants provided written informed consent. The research lasted from September 2022 through March 2023.

### 2.2 Participants

A non‐randomized convenience sampling design was followed to recruit 381 community dwelling adults aged 20 to 79 years (mean age = 46.26; SD = 15.63). The participants were grouped based on age into young adults (20–39.11 years; mean age = 28.55; SD = 6.66), middle aged adults (40–59.11 years; mean age = 47.18; SD = 5.10) and older adults (60–79.11 years; mean age = 66.29; SD = 5.02). The sample size formula *n* = z^2^pq/d^2^ determined the sample size, with Z = 1.96 at a 5% significance level, *p* = 71% based on literature (McHutchion et al. 2021), and *d* = 5%. Individuals with a history of dysphagia, neurological disorders, head and neck cancer, congenital blindness, or who are currently on non‐oral feeds were excluded from the study. The survey also excluded all healthcare professionals.

### 2.3 Development of the Survey Questionnaire

The first author developed the beta version of the survey, which included thirty‐one questions in the English language. The survey included specific inquiries under the following sections: (1) anatomy and physiology of swallowing; (2) etiology, signs and symptoms of dysphagia; and (3) assessment and management of dysphagia. In addition, the survey also probed the public's attitude and beliefs towards dysphagia and individuals with swallowing difficulties. Additionally, the study also probed the common self‐practices followed by the public to compensate for swallowing difficulties. The survey was structured with specific inquiries under these sections to obtain a holistic view of swallowing and dysphagia, encompassing its medical, social and practical dimensions. The questionnaire was written in layman's words to ensure easy readability and comprehension. Technical and medical terms were avoided. The beta version was discussed with all the researchers of the study to fine‐tune the questions. Following which, four questions were removed (three for lack of specificity and one for redundancy), resulting in a shortlist of twenty‐seven questions for further content validation.

Three Speech‐Language Pathologists (SLPs) unrelated to the study, having a minimum of five years of experience in the assessment and treatment of dysphagia, served as subject experts for content validation. The first and corresponding authors reviewed the inputs and feedback and made appropriate changes to the survey questions. Following content validation, we conducted a pilot test of the survey questions on five randomly selected non‐healthcare professionals with characteristics similar to those of the target population. All five participants described the survey questions as easy to read and comprehendible.

Five SLPs independently translated the validated English version into Kannada using standard forward translation (Wild et al. 2009). The first author then compiled the five translations and summarized the best version of each question. Two SLPs proficient in reading and writing English and Kannada performed the back translation using the parallel back translation (Sperber 2004) method. Kannada is a Dravidian language native to Karnataka, spoken by more than 4.3 billion individuals (Office of the Registrar General & Census Commissioner 2011). 66.54% of the Karnataka public, including those in Mangalore city, use the Kannada language daily (Office of the Registrar General & Census Commissioner 2011). The original questionnaire was compared to the back‐translated version by the first author of the study. Two SLPs reconciled the translations that were linguistically incorrect, incomplete and ambiguous. This method was repeated until the revised back‐translated version matched the original version. Lastly, a professional translator reviewed the linguistic content, and the corrections suggested were implemented. The Kannada version of the questionnaire was then pilot‐tested on five random non‐healthcare professionals, who reported the questionnaire as easy to read and understand.

The survey was divided into four sections: (1) demographic details; (2) knowledge about swallowing and dysphagia; (3) attitude and beliefs towards dysphagia and dysphagics (or individuals with dysphagia); and (4) strategies and practices followed to compensate for dysphagia. We used a combination of open and closed‐ended formats to obtain maximum reliable responses from the participants. Closed‐ended responses included dichotomous (Yes / No and True / False) options and forced multiple‐choice systems. Participants used the blank space below specific questions to share their thoughts on the questions with open‐ended comments.

### 2.4 Data Collection

We approached community dwellers both online and in person. We used a Google form (Google LLC, USA) to survey using the online approach. We deselected ‘collect email address’ in the Google form settings to enable more public engagement while maintaining participant anonymity. The automatic saving option in the Google form settings allowed participants to continue filling the survey from the last question (or sections) they attended. Participants could submit their responses through a Google form link kept active for eleven months (from 8 September 2022 to 1 July 2023). We also distributed hard copies of the survey questionnaire in organizational settings (colleges, banks and offices) and public locations (malls and stores) for the offline approach. Participants completed the survey either in English (Appendix 1A) or Kannada (Appendix 1B) language according to their comfort and convenience, regardless of whether the data was collected online or offline. The survey took about 15–20 min to complete.

### 2.5 Data Analysis

Responses obtained from Google form were downloaded into a Microsoft Excel spreadsheet. Data collected through offline methods were manually entered into the same spreadsheet by the second author and cross‐verified by the third author of the study. Responses of open‐ and closed‐ended questions were analyzed separately. For closed‐ended questions, responses were summarized as percentages of accuracy, calculated by dividing the total number of correct responses by the total number of responses and converting the result into a percentage. Responses to open‐ended questions were independently analyzed by the second and third authors of the study. Both researchers analyzed the data using the open content analysis procedure (Graneheim and Lundman 2004) to identify the main categories representing concepts and/or sub‐concepts of swallowing and dysphagia specific to the inquiry. Responses were categorized as ‘correct’ if the identified theme matched the specific inquiry and ‘incorrect’ if it did not match the question. In cases of disagreement between two researchers, the first author mediated and made the final decision. Similarly, for closed‐ended questions, the number of correct responses was converted into a percentage of accuracy using the same formula. Due to the nature of open‐ended questions, which allowed multiple responses per inquiry, the cumulative number of responses varied across questions. The continuous data were converted to dichotomous categories (as having good or poor knowledge; practiced or not practiced self‐management practices). This method was followed to allow testing of association of KAP with age, sex and education levels for open‐ended questions.

All statistical analysis was carried out using the Statistical Package for the Social Studies (SPSS Inc., IBM Corp, USA) software version 25. Descriptive statistics (frequency and percentage) were used to summarize the data. The chi‐square test was applied to examine associations between independent variables (sub‐groups), including age (young, middle aged and older adults), sex (male and female), and education level (graduates and non‐graduates) and participants KAP related to swallowing and dysphagia at 0.05 level.

## 3 Results

We recruited 284 (74.54%) participants offline and 97 (25.46%) participants online. 279 (73.23%) participants submitted their responses in English and 102 (26.77%) in Kannada language. The first author compiled all the survey responses into a Microsoft Excel spreadsheet for statistical analysis. We excluded nine responses (2.36%) of participants who reported being health care professionals (four nurses, two dentists, one each medical practitioner, physical therapist and SLP) from the cohort of 381 survey submissions. As a result, 372 responses were analyzed to identify the KAP related to swallowing and dysphagia.

### 3.1 Respondent Details

Table 1 summarizes participant details of the study. The participants included almost equal Males and Females (50.81% and 49.19%) and graduates and non‐graduates (46.24% and 53.76%). More than half of the participants (58.60%) were currently employed in various non‐health sectors. Overall, the demographics of all participants were equally distributed.

**TABLE 1:** Participant details of the survey (*n* = 372).

Category			*n* (%)
Age	Young adults	20–39.11	122 (32.80%)
	Middle aged adults	40–59.11	148 (39.78%)
	Older adults	60–79.11	102 (27.42%)
Sex		Male	189 (50.81%)
		Female	183 (49.19%)
Education	Non‐graduate	High school	61 (16.40%)
		College	106 (28.49%)
		Diploma	33 (8.87%)
	Graduate	Undergraduate	144 (38.71%)
		Postgraduate	25 (6.72%)
		Doctoral	3 (0.81%)
Work setting	Employed	Private	106 (28.49%)
		Self‐employed	105 (28.83%)
		Government	7 (1.88%)
	Not employed	Home maker	62 (16.67%)
		Retired	58 (15.59%)
		Student	34 (9.14%)

### 3.2 Knowledge of Swallowing and Dysphagia

Table 2 reveals the participant's overall knowledge of the close‐ended queries about swallowing and dysphagia was good. A comparison across age groups suggested that the overall knowledge of middle aged adults was highest, followed by young adults, and the overall knowledge was least among older adults. Age significantly influenced participant's knowledge about individuals with swallowing difficulties avoiding eating in public to overcome stigma [*X* ^2^(2) = 5.980; *p* = 0.05]. Sex did not significantly impact participant's knowledge related to swallowing and dysphagia at *p* > 0.05. Education levels significantly influenced participant's knowledge about saliva in chewing and swallowing [*X* ^2^(1) = 4.004; *p* = 0.045] and early intervention in regaining swallowing abilities [*X* ^2^(1) = 3.7.057; *p* = 0.008].

**TABLE 2:** Frequency distribution of responses to close‐ended questions (*n* = 372).

Probe concept	Probe question	Young adults		Middle aged adults		Older adults		Total	
		Yes/True	No/False	Yes/True	No/False	Yes/True	No/False	Yes/True	No/False
K1	Swallowing food and liquids into the lungs is ‘safe’	13 (3.49%)	**109 (29.30%)**	18 (4.84%)	**130 (34.95%)**	14 (3.76%)	**88 (23.66%)**	45 (12.10%)	**327 (87.90%)**
K2	Saliva is necessary for chewing and swallowing	**112 (30.11%)**	10 (2.69%)	**140 (37.63%)**	8 (2.15%)	**97 (26.08%)**	5 (1.34%)	**349 (93.82%)**	23 (6.18%)
K3	Dysphagia is life threatening (or dangerous to life)	**85 (22.85%)**	37 (9.95%)	**95 (25.54%)**	53 (14.25%)	**77 (20.70%)**	25 (6.72%)	**257 (69.09%)**	115 (30.91%)
K4	Swallowing ability can be regained with timely help	**115 (30.91%)**	7 (1.88%)	**139 (37.37%)**	9 (2.42%)	**98 (26.34%)**	4 (1.08%)	**352 (94.62%)**	20 (5.38%)
K5	Individuals with swallowing difficulties avoid eating in public to overcome stigma	**91 (24.46%)**	31 (8.33%)	**110 (29.57%)**	38 (10.22%)	**88 (23.66%)**	14 (3.76%)	**289 (77.69%)**	83 (22.31%)
A1	Fear choking while swallowing food	39 (10.48%)	**83 (22.31%)**	27 (7.26%)	**121 (32.53%)**	24 (6.45%)	**78 (20.97%)**	90 (24.19%)	**282 (78.81%)**
A2	Prefer eating through a pipe in your nose	20 (5.38%)	**102 (27.42%)**	47 (12.63%)	**101 (27.15%)**	19 (5.11%)	**83 (22.31%)**	86 (23.12%)	**286 (76.88%)**
A3	Follow medical advice to overcome swallowing difficulty	**116 (31.18%)**	6 (1.61%)	**142 (38.17%)**	6 (1.61%)	**99 (26.61%)**	3 (0.81%)	**357 (95.97%)**	15 (4.03%)
A4	Comfortable when someone with swallowing difficulty is eating through a pipe	47 (12.63%)	**75 (20.16%)**	65 (17.47%)	**83 (22.31%)**	**55 (14.78%)**	47 (12.63%)	167 (44.89%)	**205 (55.11%)**
A5	Ready to take help for swallowing difficulty from family during mealtime	**110 (29.57%)**	12 (3.23%)	**136 (36.56%)**	12 (3.23%)	**95 (25.54%)**	7 (1.88%)	**341 (91.67%)**	31 (8.33%)
P1	Used Internet to find answers to overcome swallowing difficulty	16 (4.30%)	**106 (28.49%)**	17 (4.57%)	**131 (35.22%)**	5 (1.34%)	**97 (26.08%)**	38 (10.22%)	**334 (89.78%)**
P2	Visited doctor for swallowing difficulty	15 (4.03%)	**107 (28.76%)**	25 (6.72%)	**123 (33.06%)**	12 (3.23%)	**90 (24.19%)**	52 (13.98%)	**320 (86.02%)**

Abbreviations: A, Attitude; K, Knowledge; P, Practice.

Bold characters indicate the highest response for the probe question of each age group.

The results of the remaining close‐ended questions and all open‐ended questions are summarized below, organized into specific sections, and responses are presented in decreasing order of frequency.

#### 3.2.1 Body Parts Involved in Swallowing

901 responses correctly identified the body parts involved in swallowing. Table S1 shows that the tongue (27.08%) and mouth (24.86%) were the most known and cheeks and hard palate (0.22%) were the least known body parts involved in swallowing. Age, sex and education levels did not significantly affect the knowledge of this enquiry.

#### 3.2.2 Medical Conditions Causing Dysphagia

58 responses were correct medical conditions leading to dysphagia. Table 3 shows that cancer of the head and neck (84%) as the leading cause and aging (0.16%) as the least known cause of dysphagia. Age, sex and education levels did not significantly affect the knowledge of this enquiry.

**TABLE 3:** Knowledge of the etiologies of dysphagia among participants (*n* = 58).

Medical conditions		Young adults	Middle aged adults	Old adults	Total
Cancer	Oral cavity	7 (14%)	3 (6%)	8 (16%)	18 (36%)
	Throat	8 (16%)	6 (12%)	4 (8%)	18 (36%)
	Stomach	2 (4%)	1 (2%)	2 (4%)	5 (10%)
	Radiation therapy	0 (0%)	1 (2%)	0 (0%)	1 (2%)
	Overall	17 (34%)	11 (22%)	14 (28%)	42 (84%)
Stroke		4 (0.65%)	3 (0.49%)	1 (0.16%)	8 (1.31%)
Neurological disease		2 (0.33%)	2 (0.33%)	3 (0.49%)	7 (1.14%)
Aging		0 (0%)	0 (0%)	1 (0.16%)	1 (0.16%)
Total		24 (41.38%)	16 (27.59%)	18 (31.03%)	58 (100%)

#### 3.2.3 The Age at Which Dysphagia due to Medical Illness Occurs Frequently

**FIGURE 1:**
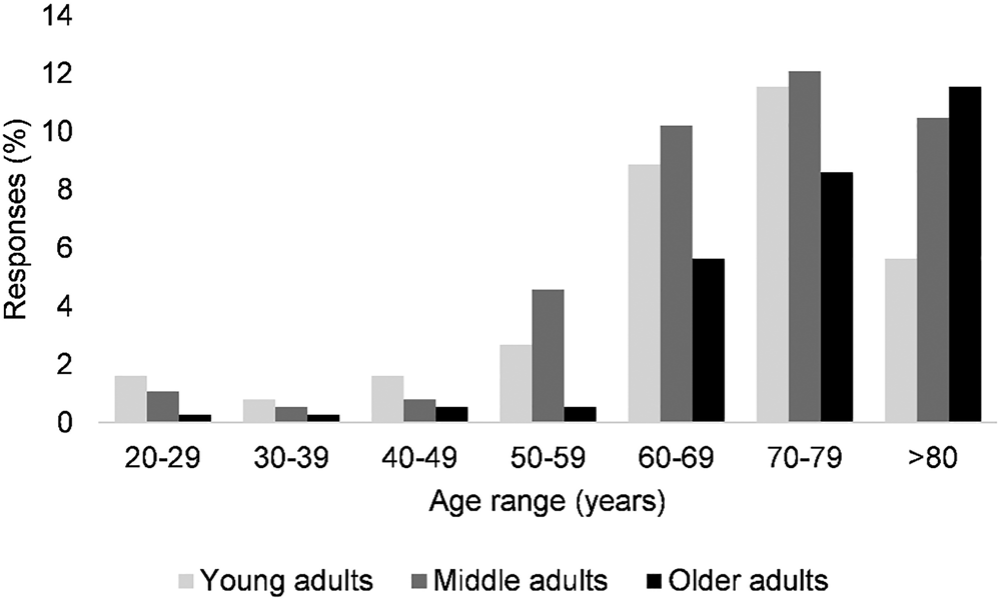
Age range perceived by the participants as being frequently associated with dysphagia.

Figure 1 depicts participant's knowledge about the age range where dysphagia due to medical illness is common. 315 (84.68%) participants reported frequent swallowing difficulties after the sixth decade of life. Age significantly influenced participants’ knowledge of this inquiry [*X* ^2^(2) = 10.093; *p* = 0.006].

#### 3.2.4 Signs of Swallowing Difficulties

151 correctly represented swallowing difficulties. Table 4 shows that choking was the most common sign and multiple swallows and drooling (0.66%) were the least common signs of dysphagia. Age, sex and education levels did not significantly affect the knowledge of this enquiry.

**TABLE 4:** Knowledge of the signs of dysphagia among participants (*n* = 151).

Sign	Young adults	Middle aged adults	Old adults	Total
Choking	17 (11.26%)	17 (11.26%)	9 (5.96%)	43 (28.48%)
Coughing	17 (11.26%)	14 (9.27%)	8 (5.30%)	39 (25.83%)
Pain while swallowing	12 (7.95%)	12 (7.95%)	8 (5.30%)	32 (21.19%)
Chewing difficulty	6 (3.97%)	7 (4.64%)	0 (0%)	13 (8.61%)
Food stuck in throat	4 (2.65%)	5 (3.31%)	3 (1.99%)	12 (7.95%)
Dry mouth	2 (1.32%)	2 (1.32%)	2 (1.32%)	6 (3.97%)
Aspiration	3 (1.99%)	0 (0%)	1 (0.66%)	4 (2.65%)
Drooling	1 (0.66%)	0 (0%)	0 (0%)	1 (0.66%)
Multiple swallows	0 (0%)	0 (0%)	1 (0.66%)	1 (0.66%)
Total	62 (41.06%)	57 (37.55%)	32 (21.19)	151 (100%)

#### 3.2.5 Frequency of Experiencing Dysphagia

317 (85.21%) participants reported never experiencing dysphagia. As shown in Table S2, 33 older adults (8.87%) reported experiencing swallowing difficulties more than once than younger and middle aged adults. Age, sex and education levels did not significantly affect the knowledge of this enquiry.

#### 3.2.6 Impact of Dysphagia on Aspects of Life

54 participants (10.29%) reported dysphagia has no consequences in an individual's life. As shown in Table S3, 174 participants (33.14%) reported dysphagia affects financial, personal, professional and social aspects of life, while 36 (6.86%) participants reported dysphagia least affects social life. Age, sex and education levels did not significantly affect the knowledge of this enquiry.

#### 3.2.7 Ramifications of Dysphagia

194 responses were correct ramifications of dysphagia. As shown in Table 5, 57 (29.38%) responses each reported weight loss and weakness as the major ramification and 6 (3.09%) responses suggested pneumonia as the least ramification of dysphagia. Age, sex and education levels did not significantly affect the knowledge of this enquiry.

**TABLE 5:** Knowledge of the ramifications of dysphagia among participants (*n* = 194).

Ramification	Young adults	Middle aged adults	Old adults	Total
Weight loss	18 (9.28%)	19 (9.79%)	20 (10.31%)	57 (29.38%)
Weakness	10 (5.15%)	25 (12.89%)	22 (11.34%)	57 (29.38%)
Nutrition deficiency	20 (10.31%)	15 (7.73%)	5 (2.58%)	40 (20.62%)
Dehydration	15 (7.73%)	7 (3.61%)	0 (0%)	22 (11.34%)
Aspiration	2 (1.03%)	2 (1.03%)	2 (1.03%)	6 (3.09%)
Pneumonia	4 (2.06%)	1 (0.52%)	1 (0.52%)	6 (3.09%)
Death	2 (1.03%)	2 (1.03%)	2 (1.03%)	6 (3.09%)
Total	71 (36.60%)	71 (36.60%)	88 (26.83%)	194 (100%)

#### 3.2.8 Tests to Assess Dysphagia

75 responses correctly named dysphagia assessment tests. As shown in Table S4, 55 (73.33%) responses suggested video‐fluoroscopy study of swallowing as the most known test and one response (1.33%) reported trial feeds as the least common test to assess dysphagia. Age, sex and education levels did not significantly affect the knowledge of this enquiry.

#### 3.2.9 Healthcare Professionals Involved With Swallowing and Dysphagia

328 responses correctly represented healthcare professionals involved with swallowing and dysphagia. As shown in Table S5, 179 (54.57%) reported Ear, Nose and Throat specialists as the primary health care professional, while 3 (0.91%) responses reported dietician as the health care professional for swallowing and dysphagia. Age, sex and education levels did not significantly affect the knowledge of this enquiry.

#### 3.2.10 Treatment Options for Dysphagia

44 responses correctly identified the treatment options for dysphagia. As shown in Table S6, 15 (34.09%) responses suggested modifying the diet as the most common treatment and 5 (11.36%) responses reported non‐oral tube as the least common treatment option for dysphagia. Age, sex and education levels did not significantly affect the knowledge of this enquiry.

### 3.3 Attitude Towards Swallowing and Dysphagia

Table 2 summarizes the participant's attitudes towards swallowing and dysphagia. As shown in the table, the participants had an overall negative attitude towards dysphagia. Middle aged adults reported the most negative attitudes towards dysphagia. Sex of the participants’ significantly influenced their comfort in seeing individuals with dysphagia take feeds via nasogastric tube [*X* ^2^(2) = 5.418; *p* = 0.05]. Age [*X* ^2^(2) = 6.902; *p* = 0.032], sex [*X* ^2^(1) = 11.048; *p* = 0.001] and education level [*X* ^2^(1) = 4.148; *p* = 0.042] significantly influenced fear of choking. Participants’ preference to take non‐oral feeds was significantly influenced by age [*X* ^2^(2) = 10.475; *p* = 0.005], sex [*X* ^2^(1) = 4.175; *p* = 0.041] and education [*X* ^2^(1) = 5.800; *p* = 0.016] levels.

### 3.4 Practices of Swallowing

Table 2 summarizes the participants’ self‐practices and behaviors to manage dysphagia. As shown in Table 2, 51 (15.50%) participants used Internet resources to find solutions for dysphagia and were significantly influenced by education [*X* ^2^(1) = 15.404; *p* = 0.000]. Furthermore, 55 (16.72%) participants visited a doctor for swallowing difficulties. Sex was significantly associated with the practice of drinking water before meals [*X* ^2^(1) = 7.046; *p* = 0.008].

#### 3.4.1 Strategies Followed to Overcome Dry Mouth

295 responses were correct strategies reported by the participants to overcome dry mouth. As shown in Table S7, 286 (96.95%) responses were drinking water to overcome dry mouth. Age, sex and education levels did not significantly affect the knowledge of this enquiry.

#### 3.4.2 Drink Water to Moisten the Mouth Before a Meal

As shown in Table S8, 187 (50.27%) participant's ‘always’ drank water before meals and 120 (32.26%) reported as ‘never’. Age [*X* ^2^(2) = 14.563; *p* = 0.001] and sex [*X* ^2^(1) = 7.046; *p* = 0.008] were significantly associated with the practice of drinking water before meals.

#### 3.4.3 Substituted Feeding by Hand to Straw or Spoon to Overcome Swallowing Difficulty

As shown in Table S8, 330 (88.71%) participants reported ‘never’ and 39 (10.48%) reported ‘always’ using a spoon or straw to overcome swallowing difficulties while feeding by hand. Sex was significantly associated with feeding by hand to spoon or straw [*X* ^2^(1) = 5.784; *p* = 0.016], but was not affected by age and education levels.

#### 3.4.4 What Do You Do When Someone Chokes on Food?

169 responses were correct practices to overcome choking on food. As shown in Table S9, 111 (39.64%) responses reported giving water to someone who chokes on food while four (1.43%) responses included doing an head extension. Age, sex and education levels did not significantly affect the knowledge of this enquiry.

#### 3.4.5 Mealtime That Required Changing Food Consistencies

As shown in Table S10, 337 (90.59%) participants reported no changes to food consistencies. Age [*X* ^2^(2) = 12.627; *p* = 0.002], sex [*X* ^2^(1) = 3.501; *p* = 0.002] and education levels [*X* ^2^(1) = 6.687; *p* = 0.010] were significantly associated with changing food consistencies among the public.

## 4 Discussion

The present study investigated KAP related to swallowing and dysphagia among the community dwellers. Participants completed a purpose‐built questionnaire comprising both open and closed‐ended questions. Overall findings suggest that community dwellers have limited knowledge, negative attitudes and unsafe self‐practices related to swallowing and dysphagia. Similar findings have been reported in recent dysphagia literature (Howells et al. 2021), where only 24.5% of the public in the United States were familiar with dysphagia and correctly identified it as an impairment only 45% of the time (Molfenter et al. 2025). Further, the overall results revealed significant differences in KAP related to swallowing and dysphagia among young, middle aged and older adults. Specifically, middle aged adults exhibited the highest level of knowledge but held more negative attitudes compared to young and older adults. In contrast, consistent with another study (Varshini et al. 2024), older adults demonstrated greater number of self‐practices to manage swallowing difficulties compared to the other two age groups. These findings are in line with existing literature reporting poor knowledge related to dysphagia among older adults, with 58.3% of older adults lacking knowledge about dysphagia and 35.5% being able to accurately define dysphagia (Jardine et al. 2021). Consequently, older adults perceive swallowing issues as a natural part of aging and overlook them. Additionally, 23.4% of older adults attribute their daily swallowing difficulties to aging (Chen et al. 2009). The specific findings of the study are discussed below in relation to existing literature and their clinical applications to SLPs and other healthcare team members involved in dysphagia care.

### 4.1 Knowledge Related to Swallowing and Dysphagia

Most participants demonstrated awareness of the body parts involved in feeding and swallowing. Notably, awareness was higher for oral structures than pharyngeal and esophageal structures. These findings have important clinical implications for SLPs’ counselling of patients and caregivers (or family). For less familiar swallowing structures, the use of visual aids such as videos and anatomical models may enhance understanding of the swallowing process. This approach can help individuals with dysphagia and their families better comprehend physiological mechanisms and engage more effectively in treatment decisions.

When asked about causes of dysphagia, participants mostly frequently identified cancer and stroke, consistent with previous literature (Bhattacharyya 2014, Grannell et al. 2001, McHutchion et al. 2021). The higher recognition of head and neck cancer may be attributed to sustained government awareness campaigns through advertisements in public spaces and social media on tobacco and alcohol packaging. We believe this serves as a model for raising awareness about other causes of dysphagia, like stroke, in low‐ and middle‐income countries.

Most participants recognized that older adults face increased medical risks that may lead to dysphagia. However, only one participant associated aging as a cause for dysphagia. Globally, population aging is emerging as a significant public health challenge, with dysphagia adding to the burden of age‐related diseases (Rajati et al. 2022, Ribeiro et al. 2023, Chang et al. 2019). Prevalence rates of swallowing difficulties range from 22.1% (Jardine et al. 2021) to 53.8% (Igarashi et al. 2019) in Western populations and 36.6% in Mangalore, India (Dodderi et al. 2024). Therefore, healthcare professionals involved in the care of older patients in India must scale their efforts and improve public awareness of dysphagia among older adults.

The most commonly identified signs of dysphagia were choking, coughing and pain while swallowing. Few participants reported other critical signs like aspiration, drooling and multiple swallows. Identification of broader signs may indicate increased self‐awareness of dysphagia, whereas failure to identify these signs may indicate limited self‐awareness of the condition. Among healthcare professionals, nearly three‐fourths identified professionals responsible for dysphagia assessment and treatment, with more than half mentioning Ear, Nose and Throat surgeons. We are not alarmed by these findings, as Ear, Nose and Throat doctors are more known among the public than SLPs for communication science disorders in India (Mandke and Bellur 2024). These findings are also consistent with existing literature that report limited awareness of the role of SLPs in the assessment and treatment of dysphagia among community dwelling older adults in India (Varshini et al. 2024). Similar findings have been reported in the United States (Molfenter et al. 2025), where 12.5% received SLP services, while 19.6% sought care from Ear, Nose and Throat surgeons (Bhattacharyya 2014). This pattern reflects the tendency to seek initial medical consultation rather than rehabilitation services, particularly in India.

Fewer than one‐fifth of participants (16.11%) reported occasional swallowing difficulties. Such experiences may represent normal variations in swallowing capacity rather than pathological conditions. Previous research indicates that even healthy adults scoring <3 on the Eating Assessment Tool‐10 may report symptoms such as frequent coughing (particularly with liquids), nasal regurgitation, choking, chewing difficulty and spontaneous saliva aspiration (Leslie and Smithard 2021). These individuals may constitute a *‘hidden population’* (Leslie and Smithard 2021), as they often do not seek medical attention for their swallowing difficulties.

Dysphagia significantly impacts multiple domains of life, including social, financial, personal and professional repercussions. In the United States, individuals with swallowing difficulties reported approximately 139 days of impact annually, including 11.6 days of leave from work (Bhattacharyya 2014). Individuals who sought treatment reported better personal and social (56.5%) and work (66.2%) lives (Bhattacharyya 2014). In the present study, although most participants recognized that timely assistance enhances safe swallowing, only one‐tenth were aware of available dysphagia treatment options. Lastly, more than three‐fourths of participants agreed that individuals with dysphagia often avoid eating at social gatherings to avoid stigmatization. Similar findings on the impact of dysphagia on social eating was reported from participants who both discussed (67.9%) and did not discuss (32.1%) their dysphagia concerns with physicians (Wilkins et al. 2007).

### 4.2 Attitude Towards Swallowing and Dysphagia

Three‐fourths of the study participants reported fear of choking while eating and swallowing. Interestingly, such fear has also been reported in adults with no risk of dysphagia (Leslie and Smithard 2021). While this perception may not affect healthy adults, it may hinder clinical assessment and dietary recommendations in clinical populations (Howells et al. 2021). Furthermore, a notable proportion of participants expressed negative attitudes, with more than three‐fourths indicating willingness to use a Ryle's tube during illness. However, over half of the participants reported discomfort when observing others using Ryle's tube.

Encouragingly, most participants demonstrated positive attitudes towards adhering to medical advice and involving their family in managing swallowing difficulties. These behaviors are encouraging for SLPs and suggest potential for enhanced compliance and greater adherence to behavioral dysphagia management. Such attitudes may help address the low adherence rates reported in dysphagia therapy literature (Krekeler et al. 2018). However, the long‐term sustainability of these positive behaviors remains uncertain.

### 4.3 Practices of Swallowing

Most participants reported drinking water as a common remedy to relieve dry mouth. However, only half of the participants *‘always’* employed this practice before meals. Prior research reports similar practice of using liquids to wash down foods among 56.7% of participants who discussed dysphagia with their physician and 43.3% of participants who did not discuss dysphagia with their physician (Wilkins et al. 2007).

A recent study reported that individuals with dysphagia sought assistance from various healthcare professionals, including primary care providers (75.3%), gastroenterologists (38.3%), oto‐rhino‐laryngologists (18.5%) and emergency room physicians (12.5%) (Adkins et al. 2020). Intriguingly, nearly half of the participants (46.3%) voluntarily chose not to discuss their symptoms with their consulting physician (Wilkins et al. 2007). Interestingly, one‐tenth of the study participants reported using internet‐based resources to address their dysphagia concerns. Given the widespread use of digital health information (Renganathan et al. 2017), further research is warranted to evaluate the quality and utilization of online resources for dysphagia care. Lastly, less than one‐tenth of participants reported using strategies like modifying food textures or using a straw to address their dysphagia. Comparable reports exist in the literature regarding various compensatory strategies, such as avoiding specific foods (71.7%), consuming smaller bites (60%) and using water to aid swallowing (56.7%) (Wilkins et al. 2007).

## 5 Clinical Implications

Based on the findings of the present study, we strongly encourage SLPs and other healthcare professionals involved in dysphagia care to increase public awareness about swallowing and dysphagia. Specifically, healthcare professionals can use the findings of the study to educate the public on knowledge of swallowing and dysphagia revealed to be weak, or primarily work on negative beliefs or misconceptions to be re‐shaped into a positive attitude. Furthermore, Basic Life Support (BLS) skills training programs, including training in the Heimlich maneuver, must be an essential life skill for the public to learn. Lastly, the public should be advised not to self‐treat swallowing problems using information listed in the internet and/or social media, but rather encouraged to seek help from healthcare professionals.

## 6 Limitations

The study has a few limitations that need to be acknowledged. The sample was drawn exclusively from an urban population, limiting rural generalisability. The second limitation pertains to the nature of the responses provided by the participants for close‐ended questions that involved binary (Yes/No) or forced multiple‐choice options. This raises the possibility of extrapolated or less nuanced responses that may not fully capture the complexity of participants’ perspectives or experiences. The third limitation of the study was treating multiple responses to open‐ended questions as individual items, potentially allowing participants to provide correct and incorrect responses. Although we considered only the correct answers, this approach may have compromised the overall reliability and validity of the study findings. The fourth limitation relates to the distribution of questions focusing on KAP regarding swallowing and dysphagia, with greater emphasis on knowledge‐related inquiries. These limitations should be considered when interpreting the results of the study and when planning future survey design research on swallowing and dysphagia.

## 7 Conclusion

Overall, this study concludes that the community dwellers demonstrated limited knowledge, negative attitudes and unsafe self‐practices and behaviors related to swallowing and dysphagia. We highly recommend that healthcare professionals develop and implement an action plan to engage the community in comprehending the fundamental aspects of swallowing and dysphagia and the importance of early self‐referral. Furthermore, we also stress the importance of training the public in the Heimlich maneuver as a first aid treatment for choking. Future studies could focus on comparing the KAP related to swallowing and dysphagia between rural and urban populations, as well as between metropolitan and non‐metropolitan cities. Furthermore, studies could investigate KAP related to swallowing and dysphagia across various etiologies, such as stroke, dementia and head and neck cancers, among others. It would be interesting to explore the differences in KAP related to swallowing and dysphagia among adults working in the healthcare sector compared to those who do not work in the healthcare sector.

## Funding

The authors have nothing to report.

## Ethics Statement

Ethical approval has been granted by the Institute Ethics Committee of Kasturba Medical College Mangalore (Approval number: IEC KMC MLR 08/2022/331).

## Consent

All participants signed the informed consent before data collection.

## Conflicts of Interest

The authors declare no conflicts of interest.

## Permission to reproduce material from other sources

No material has been reproduced from other sources

## Supporting information




**Supporting Information**: jlcd70273‐supp‐0001‐SuppMat.docx

**Supporting Information**: jlcd70273‐supp‐0002‐SuppMat.docx

## Data Availability

The datasets generated during and/or analyzed during the current study are available from the corresponding author on reasonable request.
