# Supplementary material for: Knowledge, Attitude and Practices of Swallowing and Dysphagia: A Cross‐Sectional Survey Among the Community Dwelling Adults of South India
Source: Int J Lang Commun Disord. 2026 Jun 12;61(4):e70273. doi: 10.1111/1460-6984.70273 (PMC13263545; doi:10.1111/1460-6984.70273)
Supplement: Supplementary file 1 — Supporting Information: jlcd70273‐supp‐0001‐SuppMat.docx [file JLCD-61-0-s001.docx]

**APPENDIX 1A**

**Questionnaire to assess knowledge, attitude, and practices related to swallowing and dysphagia in community dwelling adults of South India**

**Instructions: Read the questions carefully and answer all of them accordingly.**

**Section 1: Demographic details**

1. Name:
2. Age:
3. Sex:
4. Select your highest level of education:

- *High school or lower*
- *Pre-university*
- *Diploma*
- *Bachelors’ degree*
- *Masters’ degree*
- *PhD*
- *Other*

1. Currently you are employed in:

- *Government organisation*
- *Private sector*
- *Self-employed*
- *Retired*
- *Home-maker*
- *Student*

1. If you currently (or previously) worked in healthcare sector, select the field best that describes your role

- *Medical*
- *Dental*
- *Physiotherapy*
- *Occupational therapy*
- *Audiology and Speech Language Pathology*
- *Para-medical*
- *Nursing*
- *Pharmacist*
- *Veterinarian*
- *Other:*

**Section 2: Knowledge of swallowing and dysphagia**

1. Name all the human body parts involved in swallowing according to you

2. Name all the medical conditions that causes difficulty in swallowing according to you

3. Swallowing food and liquids into the lungs are ‘safe’

1. *True*
2. *False*

4. Mention all the indicators of swallowing difficulty according to you

5. Mention all the consequences of swallowing difficulty according to you

6. How frequently during breakfast/lunch/dinner have you experienced swallowing difficulty?

1. *I haven’t experienced swallowing difficulty at all*
2. *1-4 times/meal*
3. *5-10 times/meal*
4. *>10 times/meal*

7. Name all the medical test’s to check swallowing difficulty according to you

8. Mention all the treatment options for swallowing difficulty according to you

9. Name all the medical and paramedical professionals responsible for assessment and treatment of swallowing difficulty according to you

10. Saliva is necessary for chewing (mixing food in mouth) and swallowing

1. *Yes*
2. *No*

11. Select the age range where swallowing difficulty occurs most frequently due to medical conditions according to you

1. *20-29 years*
2. *30-39 years*
3. *40-49- years*
4. *50-59 years*
5. *60-69 years*
6. *70-79 years*
7. *>80 years*

12. Select the categories affected by swallowing difficulty according to you

1. *Family life*
2. *Professional life*
3. *Financial condition*
4. *Social life*
5. *None of the above*

13. Swallowing difficulty is life threatening (or dangerous to life)

1. *Yes*
2. *No*

14. Swallowing ability can be regained with timely help

1. *Yes*
2. *No*

15. Are you aware that individuals with swallowing difficulties avoid eating in family and public functions to overcome stigma

1. *Yes*
2. *No*

**Section 3: Attitude towards swallowing and dysphagia**

16. Do you fear choking while swallowing food?

1. *Yes*
2. *No*

17. Would you prefer eating through a pipe in your nose?

1. *Yes*
2. *No*

18. Would you follow medical advice to overcome swallowing difficulty?

1. *Yes*
2. *No*

19.Are you comfortable when someone with swallowing difficulty is eating through a pipe?

1. *Yes*
2. *No*

20. Are you ready to take help for swallowing difficulty from family during meal time

1. *Yes*
2. *No*

**Section 4: Practices to overcome dysphagia**

21. Do you drink water to moisten mouth and throat before starting breakfast/lunch/dinner?

1. *Always*
2. *Sometime*
3. *Never*

22. Write all the ideas to overcome dry mouth during swallowing by you?

23. Have you Googled to find answers to overcome swallowing difficulty experienced by you or for family members?

1. *Yes*
2. *No*

24. Have you visited a doctor for swallowing difficulty?

1. *Yes*
2. *No*

25. What do you do when someone chokes on food?

26. How frequently have you substituted feeding by hand to straw or spoon to overcome swallow difficulty?

1. *Always*
2. *Sometime*
3. *Never*

27. Select all the meal timings that requires you to change the food consistency by mashing it or blending it in mixer or thickening the food?

1. *Breakfast*
2. *Lunch*
3. *Evening snacks*
4. *Dinner*
5. *All four*
6. *I don’t change the food consistency*

**APPENDIX 1B**

**ದಕ್ಷಿಣ ಭಾರತದ ಜನರಲ್ಲಿ ನುಂಗುವಿಕೆ ಮತ್ತು ಡಿಸ್ಫೇಜಿಯಾಗೆ ಸಂಬಂಧಿಸಿದ ಜ್ಞಾನ, ವರ್ತನೆ ಮತ್ತು ಅಭ್ಯಾಸಗಳನ್ನು ನಿರ್ಣಯಿಸಲು ಪ್ರಶ್ನಾವಳಿ**

**ಸೂಚನೆಗಳು: ಪ್ರಶ್ನೆಗಳನ್ನು ಸರಿಯಾಗಿ ಓದಿ ಮತ್ತು ಅದಕ್ಕೆ ತಕ್ಕಂತೆ ಉತ್ತರಿಸಿ.**

**ವಿಭಾಗ 1: ವೈಯಕ್ತಿಕ ವಿವರಗಳು**

- - - 1. ಹೆಸರು
      2. ವಯಸ್ಸು
      3. ಲಿಂಗ
      4. ನಿಮ್ಮ ಉನ್ನತ ಮಟ್ಟದ ಶಿಕ್ಷಣವನ್ನು ಆಯ್ಕೆಮಾಡಿ
- *10ನೇ ತರಗತಿ ಅಥವಾ ಕಡಿಮೆ*
- *ಪಿ ಯು ಸಿ*
- *ಡಿಪ್ಲೊಮಾ*
- *ಸ್ನಾತಕ ಪೂರ್ವ ಪದವಿ*
- *ಸ್ನಾತಕೋತ್ತರ ಪದವಿ*
- *ಪಿ ಎಚ್‌ ಡಿ*
- *ಇತರ*
  - - 1. ಪ್ರಸ್ತುತ ನೀವು ಯಾವ ಉದ್ಯೋಗದಲ್ಲಿರುವಿರಿ
- *ಸರ್ಕಾರಿ ಸಂಸ್ಥೆ*
- *ಖಾಸಗಿ ವಲಯ*
- *ಸ್ವಯಂ ಉದ್ಯೋಗಿ*
- *ನಿವೃತ್ತ*
- *ಗೃಹಸ್ತಿ ಅಥವಾ ಗೃಹಕಾರ್ಯ*
- *ವಿದ್ಯಾರ್ಥಿ*
  - - 1. ನೀವು ಪ್ರಸ್ತುತ (ಅಥವಾ ಹಿಂದೆ) ಆರೋಗ್ಯ ಕ್ಷೇತ್ರದಲ್ಲಿ ಕೆಲಸ ಮಾಡುತ್ತಿದ್ದರೆ, ನಿಮ್ಮ ಪಾತ್ರವನ್ನು ವಿವರಿಸುವ ಅತ್ಯುತ್ತಮ ಕ್ಷೇತ್ರವನ್ನು ಆಯ್ಕೆಮಾಡಿ
- *ವೈದ್ಯಕೀಯ*
- *ದಂತ*
- *ಫ಼ಿಸಿಯೋ ಥೆರಪಿ*
- *ಓಕ್ಯುಪೇಶ್ನಲ್ ಥೆರಪಿ*
- *ವಾಕ್ ಮತ್ತು ಶ್ರವಣ ತಜ್ಞ*
- *ಪ್ಯಾರಾ-ಮೆಡಿಕಲ್*
- *ನರ್ಸಿಂಗ್*
- *ಫಾರ್ಮಾಸಿಸ್ಟ್*
- *ಪಶುವೈದ್ಯ*
- *ಇತರ*

**ವಿಭಾಗ 2: ನುಂಗುವಿಕೆ ಮತ್ತು ಡಿಸ್ಫೇಜಿಯಾದ ಜ್ಞಾನ**

1. ನಿಮ್ಮ ಪ್ರಕಾರ ನುಂಗುವುದಕ್ಕೆ ಬೇಕಾಗಿರುವ ಅಂಗಗಳನ್ನು ಹೆಸರಿಸಿ
2. ನಿಮ್ಮ ಪ್ರಕಾರ ನುಂಗುವ ತೊಂದರೆ ಯಾವ ವೈದ್ಯಕೀಯ ರೋಗದಿಂದ ಉಂಟಾಗುತ್ತದೆ ಎಂದು ಹೆಸರಿಸಿ
3. ಶ್ವಾಸಕೋಶಕ್ಕೆ ಆಹಾರ ಹೋಗುವುದು ಸುರಕ್ಷಿತ

- *ಸರಿ*
- *ತಪ್ಪು*

1. ನಿಮ್ಮ ಪ್ರಕಾರ ನುಂಗುವ ತೊಂದರೆಯ ಲಕ್ಷಣಗಳನ್ನು ಹೆಸರಿಸಿ
2. ನಿಮ್ಮ ಪ್ರಕಾರ ನುಂಗುವ ತೊಂದರೆಯಿಂದ ಆಗುವ ದುಷ್ಪರಿಣಾಮಗಳನ್ನು ಹೆಸರಿಸಿ
3. ನೀವು ಎಷ್ಟು ಬಾರಿ ತಿಂಡಿ/ಊಟದ ಸಮಯದಲ್ಲಿ ನುಂಗಲು ತೊಂದರೆ ಅನುಭವಿಸಿದ್ದೀರಾ?

- *ನಾನು ನುಂಗಲು ಕಷ್ಟವನ್ನು ಅನುಭವಿಸಿಲ್ಲ*
- *1-4 ಬಾರಿ ಪ್ರತಿ ಸಲ*
- *5-10 ಬಾರಿ ಪ್ರತಿ ಸಲ*
- *10 ಬಾರಿಗಿಂತ ಹೆಚ್ಚು ಪ್ರತಿ ಸಲ*

1. ನುಂಗುವ ತೊಂದರೆ ಪರಿಶೀಲಿಸುವ *ವೈದ್ಯಕೀಯ* ಪರೀಕ್ಷೆಗಳನ್ನು ಹೆಸರಿಸಿ
2. ನಿಮ್ಮ ಪ್ರಕಾರ ನುಂಗುವ ತೊಂದರೆಯ ಚಿಕಿತ್ಸೆಗಳನ್ನು ಹೆಸರಿಸಿ
3. ನಿಮ್ಮ ಪ್ರಕಾರ ಯಾವ ವೈದ್ಯಕೀಯ ಮತ್ತು ಅರೆವೈದ್ಯಕೀಯ ಸಿಬಂದಿಯು ನುಂಗುವ ತೊಂದರೆಯ ಪರೀಕ್ಷೆ ಮತ್ತು ಚಿಕಿತ್ಸೆಯಲ್ಲಿ ಕಾರ್ಯ ನಿರ್ವಹಿಸುತ್ತಾರೆ?
4. ಆಹಾರವನ್ನು ಅಗಿಯಲು ಹಾಗು ಮಿಶ್ರಣ ಮಾಡಲು ಎಂಜಿಲು ಅಗತ್ಯ

- *ಹೌದು*
- *ಇಲ್ಲ*

1. ನಿಮ್ಮ ಪ್ರಕಾರ ಯಾವ ವಯಸ್ಸಲ್ಲಿ ಆರೋಗ್ಯದ ತೊಂದರೆಯಿಂದ ನುಂಗುವ ತೊಂದರೆ ಹೆಚ್ಚಾಗಿ ಕಂಡುಬರುತ್ತದೆ

- *20-29 ವರ್ಷ*
- *30-39 ವರ್ಷ*
- *40-49- ವರ್ಷ*
- *50-59 ವರ್ಷ*
- *60-69 ವರ್ಷ*
- *70-79 ವರ್ಷ*
- *80 ವರ್ಷಕ್ಕಿಂತ ಹೆಚ್ಚು*

1. ನಿಮ್ಮ ಪ್ರಕಾರ ನುಂಗುವ ತೊಂದರೆ ಜೀವನದ ಯಾವ ಅಂಶಗಳಲ್ಲಿ ದುಷ್ಪರಿಣಾಮ ಬೀರುತ್ತದೆ

- *ಕೌಟುಂಬಿಕ ಜೀವನ*
- *ವೃತ್ತಿಪರ ಜೀವನ*
- *ಹಣಕಾಸಿನ ಸ್ಥಿತಿ*
- *ಸಾಮಾಜಿಕ ಜೀವನ*
- *ಮೇಲಿನ ಎಲ್ಲವೂ*
- *ಮೇಲಿನ ಯಾವುದೂ ಅಲ್ಲ*

1. ನುಂಗುವ ತೊಂದರೆ ಜೀವಕ್ಕೆ ಅಪಾಯ

- *ಹೌದು*
- *ಇಲ್ಲ*

1. ಸಮಯಕ್ಕೆ ಸರಿಯಾದ ಸಹಾಯದಿಂದ ನುಂಗುವ ಶಕ್ತಿಯನ್ನು ಮರಳಿ ಪಡೆಯಬಹುದು

- *ಹೌದು*
- *ಇಲ್ಲ*

1. ನುಂಗುವ ತೊಂದರೆಉಳ್ಳವರು ಮುಜುಗರಕ್ಕೆ ಒಳಗಾಗದಿರಲು ಸಾಮಾಜಿಕ ಸಭೆ ಸಮಾರಂಗಳಲ್ಲಿ ತಿನ್ನುವುದಿಲ್ಲ ಎಂದು ನಿಮಗೆ ತಿಳಿದಿದೆಯಾ

- *ಹೌದು*
- *ಇಲ್ಲ*

**ವಿಭಾಗ 3: ನುಂಗುವಿಕೆ ಮತ್ತು ಡಿಸ್ಫೇಜಿಯಾ ಕಡೆಗೆ ವರ್ತನೆ**

1. ನೀವು ಆಹಾರ ನುಂಗುವಾಗ ಉಸಿರು ಕಟ್ಟುತ್ತದೆ ಎಂಬ ಭಯವಿದೆಯೇ?

- *ಹೌದು*
- *ಇಲ್ಲ*

1. ನೀವು ಮೂಗಿನ ಪೈಪ್ ಮೂಲಕ ಆಹಾರ ಸೇವಿಸಲು ಬಯಸುತ್ತೀರಾ?

- *ಹೌದು*
- *ಇಲ್ಲ*

1. ನುಂಗುವ ತೊಂದರೆಯನ್ನು ನಿವಾರಿಸಲು ವೈದ್ಯರ ಸಲಹೆಯನ್ನು ನೀವು ಪಾಲಿಸುವಿರ?

- *ಹೌದು*
- *ಇಲ್ಲ*

1. ಯಾರಾದರು ಮೂಗಿನ ಪೈಪ್ ಮೂಲಕ ಆಹಾರ ಸೇವಿಸಿದರೆ ನಿಮಗೆ ಅಸಹ್ಯ (ಮುಜುಗರ) ಅನಿಸುತ್ತದೆಯೇ?

- *ಹೌದು*
- *ಇಲ್ಲ*

1. ನುಂಗುವ ತೊಂದರೆಯನ್ನು ನಿವಾರಿಸಲು ಊಟದ ಸಮಯದಲ್ಲಿ ಕುಟುಂಬದವರಿಂದ ಸಹಾಯ ಪಡೆಯಲು ನೀವು ಸಿದ್ಧರಿದ್ಧೀರಾ?

- *ಹೌದು*
- *ಇಲ್ಲ*

**ವಿಭಾಗ 4: ನುಂಗುವ ತೊಂದರೆಯನ್ನು ನಿವಾರಿಸುವ ಅಭ್ಯಾಸಗಳು**

1. ನೀವು ಊಟ/ತಿಂಡಿ ಮಾಡುವ ಮುನ್ನ ಬಾಯಿ ಮತ್ತು ಗಂಟಲನ್ನು ನೀರು ಕುಡಿದು ಒದ್ದೆ ಮಾಡಿಕೊಳ್ಳುತ್ತೀರಾ?

- *ಯಾವಾಗಲೂ*
- *ಕೆಲವೊಮ್ಮೆ*
- *ಇಲ್ಲವೇ ಇಲ್ಲ*

1. ಜಗಿಯುವಾಗ ಅಥವಾ ನುಂಗುವಾಗ ಒಣ ಬಾಯಿ ತೊಂದರೆ ಬಗೆಹರಿಸಲು ನೀವು ಏನು ಮಾಡುತ್ತೀರಾ?
2. ನೀವು ಅಥವಾ ನಿಮ್ಮ ಕುಟುಂಬದವರ ನುಂಗುವ ತೊಂದರೆ ನಿವಾರಿಸಲು ಗೂಗಲ್ ಬಳಸಿದ್ದೀರಾ?

- *ಹೌದು*
- *ಇಲ್ಲ*

1. ನೀವು ನುಂಗುವ ತೊಂದರೆ ನಿವಾರಣೆಗಾಗಿ ವೈದ್ಯರನ್ನು ಭೇಟಿಯಾಗಿದ್ದೀರಾ?

- *ಹೌದು*
- *ಇಲ್ಲ*

1. ಯಾರಿಗಾದರೂ ನುಂಗುವಾಗ ಉಸಿರು ಕಟ್ಟಿದರೆ ನೀವು ಏನು ಮಾಡುತ್ತಿರಾ?
2. ನುಂಗುವ ತೊಂದರೆಯನ್ನು ನಿವಾರಿಸಲು ಕೈಯಿಂದ ತಿನ್ನುವ ಬದಲು ಚಮಚವನ್ನು ಎಷ್ಟು ಸಾರಿ ಉಪಯೋಗಿಸಿದ್ದೀರಾ?

- *ಯಾವಾಗಲೂ*
- *ಕೆಲವೊಮ್ಮೆ*
- *ಇಲ್ಲವೇ ಇಲ್ಲ*

1. ಯಾವ ಊಟದ ಸಮಯದಲ್ಲಿ ನೀವು ಆಹಾರವನ್ನು ಕೈಯಿಂದ (ಪದೇ ಪದೇ) ಹಿಸುಕಿ ಅಥವಾ ಮಿಕ್ಸಿಯಲ್ಲಿ ಆಹಾರದ ಸ್ಥಿರತೆಯನ್ನು (ಹದವನ್ನು) ಬದಲಾಯಿಸುತ್ತೀರಾ?

- *ಬೆಳಿಗ್ಗೆ ತಿಂಡಿ*
- *ಮಧ್ಯಾನದ ಊಟ*
- *ಸಂಜೆ*
- *ರಾತ್ರೆ ಊಟ*
- *ಮೇಲಿನ ಎಲ್ಲಾ ನಾಲ್ಕು*
- *ನಾನು ಆಹಾರದ ಸ್ಥಿರತೆಯನ್ನು ಬದಲಾಯಿಸುವುದಿಲ್ಲ*
