# Supplementary material for: Knowledge, Attitude and Practices of Swallowing and Dysphagia: A Cross‐Sectional Survey Among the Community Dwelling Adults of South India
Source: Int J Lang Commun Disord. 2026 Jun 12;61(4):e70273. doi: 10.1111/1460-6984.70273 (PMC13263545; doi:10.1111/1460-6984.70273)
Supplement: Supplementary file 2 — Supporting Information: jlcd70273‐supp‐0002‐SuppMat.docx [file JLCD-61-0-s002.docx]

**Table S1.** Knowledge of the anatomical regions of oral, pharyngeal, and oesophageal phase of swallowing among participants (n=901)

| Phase | Parts | Young adults | Middle aged adults | Old adults | Total |
| --- | --- | --- | --- | --- | --- |
| Oral | Tongue | 78 (8.66%) | 87 (9.66%) | 79 (8.77%) | 244 (27.08%) |
|  | Mouth | 74 (8.21%) | 87 (9.66%) | 63 (6.99%) | 224 (24.86%) |
|  | Teeth | 31 (3.44%) | 38 (4.22%) | 15 (1.66%) | 84 (9.32%) |
|  | Lips | 2 (0.22%) | 5 (0.55%) | 3 (0.33%) | 10 (1.11%) |
|  | Soft palate | 3 (0.33%) | 1 (0.11%) | 0 (0%) | 4 (0.44%) |
|  | Jaw | 1 (0.11%) | 2 (0.22%) | 1 (0.11%) | 4 (0.44%) |
|  | Hard palate | 1 (0.11%) | 1 (0.11%) | 0 (0%) | 2 (0.22%) |
|  | Cheeks | 1 (0.11%) | 0 (0%) | 1 (0.11%) | 2 (0.22%) |
| Pharyngeal | Pharynx | 67 (7.44%) | 75 (8.32%) | 50 (5.55%) | 192 (21.31%) |
|  | Larynx | 5 (0.55%) | 4 (0.44%) | 0 (0%) | 9 (1.00%) |
|  | Epiglottis | 3 (0.33%) | 3 (0.33%) | 1 (0.11%) | 7 (0.78%) |
| Oesophageal | Oesophagus | 44 (4.88%) | 24 (2.66%) | 7 (0.78%) | 75 (8.32%) |
|  | Stomach | 9 (1.00%) | 14 (1.55%) | 21 (2.33%) | 44 (4.88%) |
| Total | | 319 (35.41%) | 341 (37.85%) | 241 (26.75%) | 901 (100%) |

**Table S2.** Knowledge of the frequency of experiencing dysphagia among participants

| Category | Young adults | Middle aged adults | Old adults | Total |
| --- | --- | --- | --- | --- |
| Never | 112 (30.11%) | 138 (37.10%) | 69 (18.55%) | 317 (85.21%) |
| 1-5 times/day | 10 (2.69%) | 10 (2.69%) | 21 (5.65%) | 43 (11.57%) |
| 6-10 times/day | 0 (0%) | 0 (0%) | 6 (1.61%) | 6 (1.61%) |
| >10 times/day | 0 (0%) | 0 (0%) | 6 (1.61%) | 6 (1.61%) |
| Total | 122 (32.80%) | 148 (39.78%) | 102 (27.42%) | 372 (100%) |

**Table S3.** Knowledge of the consequences of dysphagia on different aspects of life among participants (n=525)

| Category | Young adults | Middle aged adults | Old adults | Total |
| --- | --- | --- | --- | --- |
| Financial condition | 26 (4.95%) | 55 (38.73%) | 22 (36.67%) | 103 (19.62%) |
| Personal life | 35 (6.67%) | 38 (26.76%) | 15 (25.00%) | 88 (16.76%) |
| Professional life | 21 (4.00%) | 36 (25.35%) | 13 (21.67%) | 70 (13.33%) |
| Social life | 14 (2.67%) | 12 (8.45%) | 10 (16.67%) | 36 (6.86%) |
| All of the above | 174 (33.14%) | 0 (0%) | 0 (0%) | 174 (33.14%) |
| None of the above | 53 (16.41%) | 1 (0.70%) | 0 (0%) | 54 (10.29%) |
| Total | 323 (61.52%) | 142 (27.05%) | 60 (11.43%) | 525 (100%) |

**Table S4.** Knowledge of the tests for dysphagia assessment among participants (n=75)

| Test | Young adults | Middle aged adults | Old adults | Total |
| --- | --- | --- | --- | --- |
| VFSS | 20 (26.67%) | 22 (29.33%) | 13 (17.33%) | 55 (73.33%) |
| Endoscopy | 3 (4%) | 8 (10.67%) | 4 (5.33%) | 15 (20%) |
| Manometry | 2 (2.67%) | 0 (0%) | 0 (0%) | 2 (2.67%) |
| OPME | 1 (1.33%) | 0 (0%) | 1 (1.33%) | 2 (2.67%) |
| Trial feeds | 0 (0%) | 0 (0%) | 1 (1.33%) | 1 (1.33%) |
| Total | 26 (34.67%) | 30 (40%) | 19 (25.33%) | 75 (100%) |
| VFSS – video fluoroscopy study of swallowing  OPME – oral peripheral speech mechanism examination | | | | |

**Table S5.** Knowledge of the health-care team involved with swallowing and dysphagia among participants (n=328)

| Health-care professional | Young adults | Middle aged adults | Old adults | Total |
| --- | --- | --- | --- | --- |
| ENT | 52 (15.85%) | 79 (24.09%) | 48 (14.63%) | 179 (54.57%) |
| Physician | 25 (7.62%) | 36 (10.98%) | 19 (5.79%) | 80 (24.39%) |
| SLPs | 12 (3.66%) | 4 (1.22%) | 6 (1.83%) | 22 (6.71%) |
| Gastrologist | 5 (1.52%) | 6 (1.83%) | 4 (1.22%) | 15 (4.57%) |
| Medical (un-specified) | 2 (0.61%) | 7 (2.13%) | 3 (0.91%) | 12 (3.66%) |
| Physiotherapists | 3 (0.91%) | 1 (0.30%) | 3 (0.91%) | 7 (2.13%) |
| Neurologist / Neuro-surgeon | 0 (0%) | 3 (0.91%) | 2 (0.61%) | 5 (1.52%) |
| Nurse | 1 (0.30%) | 1 (0.30%) | 3 (0.91%) | 5 (1.52%) |
| Dietician | 3 (0.91%) | 0 (0%) | 0 (0%) | 3 (0.91%) |
| Total | 103 (31.40%) | 137 (41.77%) | 88 (26.83%) | 328 (100%) |
| ENT - Ear Nose & Throat  SLPs - Speech Language Pathologists | |  |  |  |

**Table S6.** Knowledge of the treatment options for dysphagia among participants (n=44)

| Treatment | Young adults | Middle aged adults | Old adults | Total |
| --- | --- | --- | --- | --- |
| Modify diet | 6 (13.64%) | 8 (18.18%) | 1 (2.27%) | 15 (34.09%) |
| Exercise | 4 (9.09%) | 7 (15.91%) | 3 (6.82%) | 14 (31.82%) |
| Surgical | 5 (11.36%) | 3 (6.82%) | 2 (4.55%) | 10 (22.73%) |
| Non-oral tube | 1 (2.27%) | 3 (6.82%) | 1 (2.27%) | 5 (11.36%) |
| Total | 16 (36.36%) | 21 (47.73%) | 7 (15.91%) | 44 (100%) |

**Table S7**. Stratergies to overcome dry mouth among participants (n=295)

| Stratergies | Young adults | Middle aged adults | Old adults | Total |
| --- | --- | --- | --- | --- |
| Drinking water | 90 (30.51%) | 119 (40.34%) | 77 (26.10%) | 286 (96.95%) |
| Eating moist or liquid items | 2 (0.68%) | 3 (1.02%), | 4 (1.36%) | 9 (3.05%) |
| Total | 92 (31.19%) | 122 (41.36%) | 81 (27.45%) | 295 (100%) |

**Table S8.** Practices of swallowing and dysphagia among participants (n=372)

| Practice | Response | Young adults | Middle aged adults | Old adults | Total |
| --- | --- | --- | --- | --- | --- |
| Drink water before meals | Always | 62 (16.67%) | 62 (16.67%) | 63 (16.94%) | 187 (50.27%) |
|  | Sometime | 25 (6.72%) | 22 (5.91%) | 18 (4.84%) | 65 (17.47%) |
|  | Never | 35 (9.41%) | 64 (17.20%) | 21 (5.65%) | 120 (32.26%) |
|  | Total | 122 (32.80%) | 148 (39.78%) | 102 (27.42%) | 372 (100%) |
| Use straw / spoon instead of hands | Always | 17 (4.57%) | 14 (3.76%) | 8 (2.15%) | 39 (10.48%) |
|  | Sometime | 3 (0.81%) | 0 (0%) | 0 (0%) | 3 (0.81%) |
|  | Never | 102 (27.42%) | 134 (36.02%) | 94 (25.27%) | 330 (88.71%) |
|  | Total | 122 (32.80%) | 148 (39.78%) | 102 (27.42%) | 372 (100%) |

**Table S9.** Practices followed by participants when someone chokes on food (n=280)

| Strategy | Young adults | Middle aged adults | Old adults | Total |
| --- | --- | --- | --- | --- |
| Give water | 38 (13.57%) | 50 (17.86%) | 23 (8.21%) | 111 (39.64%) |
| Pat the back | 17 (6.07%) | 23 (8.21%) | 21 (7.50%) | 61 (21.79%) |
| Call for medical help | 16 (5.71%) | 18 (6.43%) | 5 (1.79%) | 39 (13.93%) |
| Pat the head | 13 (4.64%) | 13 (4.64%) | 12 (4.29%) | 38 (13.57%) |
| Heimlich's manoeuvre | 11 (3.93%) | 9 (3.21%) | 2 (0.71%) | 22 (7.86%) |
| Coughing | 1 (0.36%) | 2 (0.71%) | 2 (0.71%) | 5 (1.79%) |
| Head extension | 1 (0.36%) | 1 (0.36%) | 2 (0.71%) | 4 (1.43%) |
| Total | 97 (34.64%) | 116 (41.43%) | 67 (23.93) | 280 (100%) |

**Table S10.** Mealtime requiring changing food consistencies among participants (n=372)

| Mealtime | Young adults | Middle aged adults | Old adults | Total |
| --- | --- | --- | --- | --- |
| I don’t change consistencies | 118 (31.72%) | 134 (36.02%) | 85 (22.85%) | 337 (90.59%) |
| Breakfast | 1 (0.27%) | 5 (1.34%) | 2 (0.54%) | 8 (2.15%) |
| Lunch | 2 (0.54%) | 2 (0.54%) | 5 (1.34%) | 9 (2.42%) |
| Dinner | 0 (0%) | 7 (1.88%) | 3 (0.81%) | 10 (2.69%) |
| Breakfast, lunch & dinner | 1 (0.27%) | 0 (0%) | 7 (1.88%) | 8 (2.15%) |
| Total | 122 (32.80%) | 148 (39.78%) | 102 (27.42%) | 372 (100%) |
